# Gaussian Light Model in Brightfield Optical Projection Tomography

**DOI:** 10.1038/s41598-019-50469-6

**Published:** 2019-09-26

**Authors:** Olli Koskela, Toni Montonen, Birhanu Belay, Edite Figueiras, Sampsa Pursiainen, Jari Hyttinen

**Affiliations:** 1 Faculty of Medicine and Health Technology and BioMediTech Institute, Tampere University, Tampere, 33014 Finland; 2 HAMK Smart Research Unit, Häme University of Applied Sciences, Hämeenlinna, 13100 Finland; 3 Champalimaud Research, Champalimaud Foundation, Lisbon, 1400-038 Portugal; 4 Faculty of Information Technology and Communication Sciences, Tampere University, Tampere, 33014 Finland

**Keywords:** Biomedical engineering, Imaging and sensing, Imaging techniques

## Abstract

This study focuses on improving the reconstruction process of the brightfield optical projection tomography (OPT). OPT is often described as the optical equivalent of X-ray computed tomography, but based on visible light. The detection optics used to collect light in OPT focus on a certain distance and induce blurring in those features out of focus. However, the conventionally used inverse Radon transform assumes an absolute focus throughout the propagation axis. In this study, we model the focusing properties of the detection by coupling Gaussian beam model (GBM) with the Radon transform. The GBM enables the construction of a projection operator that includes modeling of the blurring caused by the light beam. We also introduce the concept of a stretched GBM (SGBM) in which the Gaussian beam is scaled in order to avoid the modeling errors related to the determination of the focal plane. Furthermore, a thresholding approach is used to compress memory usage. We tested the GBM and SGBM approaches using simulated and experimental data in mono- and multifocal modes. When compared with the traditionally used filtered backprojection algorithm, the iteratively computed reconstructions, including the Gaussian models GBM and SGBM, provided smoother images with higher contrast.

## Introduction

The imaging of samples in the mesoscopic size range of few millimeters to a centimeter is important for understanding the processes of a biological system, such as embroyos, organs or organoids of a small animal. One prominent imaging technique for imaging these mesoscopic samples natively in 3D is optical projection tomography^1,2^ (OPT), which has been successfully applied in the examination of zebrafish^3–5^, gene expression^6–8^, small organs^9–12^, tissue structures^13–16^, cells^17–19^ and hydrogels^20,21^. OPT can be used also to complement selective plane illumination microscopy^3,22^. To further enhance the information content acquired from OPT, the focus of this study is the inverse problem of reconstructing the sample from the image data.

In brightfield OPT, a wide-spectrum light beam propagates through a semi-transparent sample and projection images (shadowgrams) are recorded from a number of angles around the sample. OPT could be described as the optical equivalent of X-ray computed tomography (CT), and the associated analytic inversion algorithm of X-ray CT, filtered backprojection (FBP), has been used to reconstruct brightfield OPT images with reasonable success. There is, however, one major difference between X-ray CT and OPT concerning the detected ray geometry. The X-rays that penetrate the sample are considered to be straight lines but, in contrast, the focusing objectives in the OPT detection path shape the light beam to a Gaussian bell-shaped intensity profile^23–25^. The Gaussian shape has a certain focal distance, and in the projection images, blurring is induced into particles depending on their distance from the focus. This blurring is further seen in a decrease of radial resolution in the reconstructions. Hence, this paper focuses on incorporating the Gaussian shape, i.e., the Gaussian beam model (GBM), into the forward model of brightfield OPT with the aim of increasing the reconstruction accuracy through inversion of this enhanced model. The combined forward model is a nested convolution of type $y= {\mathcal R} \circ { {\mathcal B} }_{K}(\,\cdot \,)$ where inner functional ${ {\mathcal B} }_{K}$ describes the blurring. In standard Radon transform there is no blurring and ${ {\mathcal B} }_{K}={\rm{id}}$.

The blurring properties of the objective can be parametrized using numerical aperture (NA)^26^. The NA describes a trade-off between lateral resolution and longitudinal resolution and is directly related to the Rayleigh range of the imaging system, which describes the elongation of the Gaussian shape. In our analysis, blurring is considered to be a continuous function, symmetric to both sides of the focal distance, and GBM is represented by equation $K(\tau ,{z}_{L})=\sqrt{2/(\pi w({z}_{L}))}\exp (\,-\,2{\tau }^{2}/w({z}_{L}))$ with *w*(*z*_*L*_) = *w*_0_(1 + ((*z*_*L*_ − *z*_0_)/*z*_r_)^2^) where NA and Rayleigh range are related through NA = *w*_0_/*z*_r_. This approach does not consider the focality as a discrete value as commonly used depth-of-field (defined as twice the Rayleigh range^1^). Traditionally, objectives with low NA are used in OPT to cover at least half of the sample into depth-of-field at once. Long depth-of-field attempts to create conditions as close as possible for the assumed detection of straight lines, but also limits the lateral resolution.

Previously, decreasing the effects of the Gaussian beam shape have also been studied using image post-processing^2,27,28^, Fourier beam propagation with regularized inversion^29^, and multifocal acquisition^19,30–32^. In multifocal acquisition, several projection images are taken from each imaging angle and then fused into a single image. The fusion algorithm is chosen so that the focal parts should be preserved in the fused image. A practical implementation of multifocal acquisition is helical scanning^3,33^. In this study, multifocal acquisition is considered and compared with the results of the GBM.

Regarding fluorescence OPT, which detects the emission of excited fluorescent molecules instead of attenuation shadowgrams, several approaches to counter the blurring of particles in the reconstruction have been published. These include modified FBP and probabilistic inversion with weights based on light properties^34,35^. In addition, research on fluorescent OPT reconstruction problem includes the deconvolution of reconstructions using point spread function^36^ and adaptation of fluorescent X-ray reconstruction method^37^. Furthermore, the GBM has already been successfully applied to fluorescence OPT^38^. In contrast to brightfield OPT, which is the transmission of light through a domain, fluorescence is an emission-sources inside a domain problem, and therefore motivating a validation study in the brightfield case.

When coupled with the Radon transform, the GBM enables both the focal and blurred parts of a detected light beam to be included into a single projection operator^38^. As a result, the projection data can be used for an extended angular aperture. Consequently, the GBM can be expected to improve the reconstruction quality when compared with the inversion of the plain Radon transform. The incorporation of the GBM, however, leads to increased memory consumption and longer computation times during the inversion stage. Furthermore, as a deblurring method, the GBM-based inversion approach can be sensitive to measurement inaccuracies, such as focusing errors.

To decrease the consumption of memory, we used a thresholding approach where low values of beam intensity profile are set to zero based on the on-axis value along the propagation, i.e., the profile is enveloped. Furthermore, we introduce the concept of a stretched Gaussian beam model (SGBM), where the Gaussian beam is scaled in order to avoid the modeling errors related to the error of positioning the sample’s center of rotation in the focal plane. Scaling is achieved by multiplying the Rayleigh range *z*_*r*_ with a constant we call strecthing constant *c*_*S*_, i.e., ${z}_{{\rm{r}}}\mapsto {c}_{S}{z}_{{\rm{r}}}$, while keeping *w*_0_ in its original value. Analysis on choosing *c*_*S*_ is discussed in the Methods section. Another essential computational improvement in using the SGBM approach is that it allows a significant reduction in the memory consumption when compared with the GBM due to the faster decay of the off-axis values. For computing a reconstruction with the GBM or SGBM, we used a steepest descending minimization method that has already been described in our previous work^39^. The minimization is regularized with total variation (TV)^40^ to increase the robustness of the computation. The use of the GBM and SGBM in the reconstruction process was tested in simulated and experimental studies using both mono- and multifocal projection modes. The reconstructions are compared with standard FBP reconstructions.

**Figure 1:**
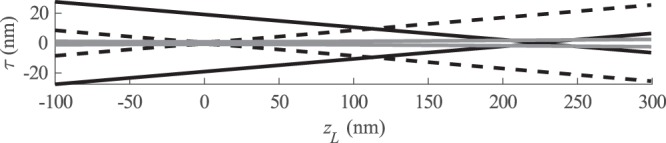
Illustration of GBM light propagation along axis *z*_*L*_. Dashed line: a GBM beam with focus at *z*_*L*_ = 0. Solid black line: a true Gaussian imaging beam with unknown focal offset. Gray line: SGBM beam, centered at *z*_*L*_ = 0, has Gaussian form and the same beam waist as GBM, but does not underweigh the values at *z*_*L*_ = 0 as much as misplaced GBM.

Figure 1 illustrates the different properties of GBM and SGBM imaging in 2D plane. Depicted with a dashed line, the ideal shape of detected GBM light has a Gaussian shape, centered at the focal point. In practice, however, plain hydrogel or cell culturing samples have no known point in the center to focus on. For this reason, it is likely that even an experienced technician would be unable to place the sample center into the focal point, and thus induces a modeling error in the direction of propagation, depicted in the figure with a solid black line. Misplacement orthogonal to propagation can be corrected easier during the imaging^41^ or by processing projection data before reconstruction^42–45^. However, the light model requires center of rotation to be at focal point in both coordinates. An SGBM beam (marked in the figure with gray solid color) can be interpreted in two ways: firstly, the physical relation is with Rayleigh limit, which is elongated without altering the beam width at focal point; and secondly, in a numerical sense, GBM and SGBM can be thought of as a weighting of the Radon transform beam, where the SGBM is thus a balance of Gaussian details and the plain Radon transform.

To analyze the performance of GBM and SGBM, we used both simulated and experimentally acquired data. In the simulations, we computed monofocal data with different focal offsets and also multifocal data. Reconstructions were computed with standard FBP, and iterative solution TV with GBM and SGBM, where different strecthing constant were applied when using SGBM. The experimental data was imaged from two samples: one including sparse population of beads and the other a zebrafish embryo which is a large continous object. In both samples, the base volume was a hydrogel suspension. Reconstructions from experimental data were computed with FBP, GBP and SGBM with stretching constant 5. In both simulated and experimental multifocal cases, the multifocal projections were flattened into single all-in-focus projections before reconstructing. The experiments in this work are summarized in Table 1 and labeled with letteres (A)–(X). In total, we present ten reconstructions from monofocal, simulated data, (A)–(J); four reconstructions from multifocal, simulated data, (K)–(N); four reconstructions from monofocal zebrafish data, (O)–(R); and five reconstructions from multifocal bead data, (S)–(X), one of which, (X), is monofocal by using only one focal plane.

**Table 1:** The identifiers and descriptions of the reconstructions.

ID	Focal Offset (s) (*μ*m)	Algorithm	Gaussian model	*β*_1_	*β*_2_	Data acquisition mode
(A)	0	FBP				monofocal, synthetic
(B)	0	TV	GBM: *K*_1_	1E-10	1E-10	
(C)	75	FBP				
(D)	75	TV	GBM: *K*_1_	1E-10	1E-10	
(E)	225	FBP				
(F)	225	TV	GBM: *K*_1_	1E-8	1E-10	
(G)	225	TV	SGBM: *K*_5_	1E-8	1E-10	
(H)	225	TV	SGBM: *K*_10_	1E-8	1E-10	
(I)	225	TV	SGBM: *K*_20_	1E-8	1E-10	
(J)	225	TV	SGBM: *K*_*δ*_	1E-8	1E-10	
(K)	−300, 0, 300	FBP				multifocal, synthetic
(L)	−300, 0, 300	TV	GBM^*^: ${K}_{1}^{\ast }$	1E-10	1E-10	
(M)	−300, 0, 300	TV	SGBM: *K*_5_	1E-10	1E-10	
(N)	−300, 0, 300	TV	SGBM: *K*_*δ*_	1E-10	1E-10	
(O)	Not specified	FBP				monofocal, zebrafish embryo
(P)	Not specified	TV	GBM: *K*_1_	1E-8	1E-10	
(Q)	Not specified	TV	SGBM: *K*_5_	1E-8	1E-10	
(R)	Not specified	TV	SGBM: *K*_*δ*_	1E-8	1E-10	
(S)	Not specified	FBP				multifocal, beads
(T)	Not specified	TV	GBM^*^: ${K}_{1}^{\ast }$	1E-8	1E-10	
(U)	Not specified	TV	GBM^*^: *K*_5_	1E-8	1E-10	
(V)	Not specified	TV	GBM^*^: *K*_*δ*_	1E-8	1E-10	
(X)	Not specified	FBP				monofocal, beads

## Results

### Results with simulated, mono- and multifocal data

Concerning the simulated experiments (A)–(J), we concentrated on the effect of focal offset, i.e., the unknown distance of focal point from the center of the sample. The effect was analyzed by simulating projection data with different offset and comparing the reconstructions of inverse methods that assumed no offset in the projection data. The forward model in simulations was a GBM without the hard thresholding of the beam.

**Figure 2:**
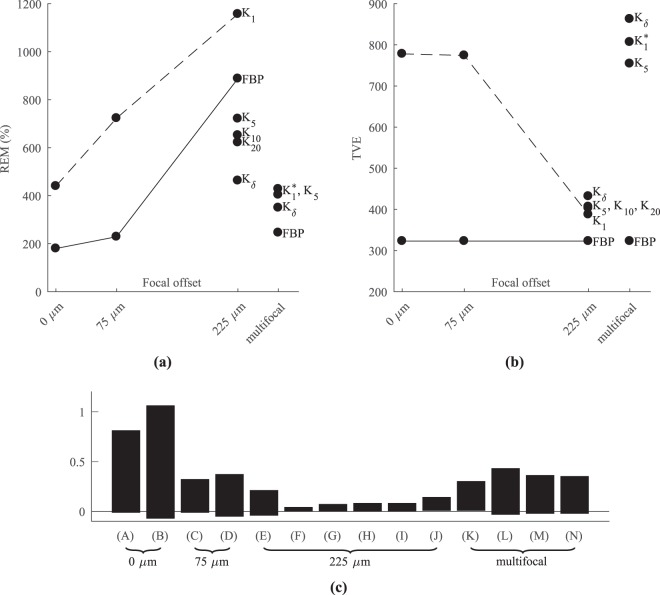
Scatter plot of (**a**) REM and (**b**) TVE values of the reconstructions (A)-(N); and (**c**) a bar chart showing the respective dynamical ranges. The REM values of the SGBM reconstructions are lower than GBM *K*_1_ and TVE values at the same level. Controversially, with multifocal data, REM values are relatively low but TVE values high.

**Figure 3:**
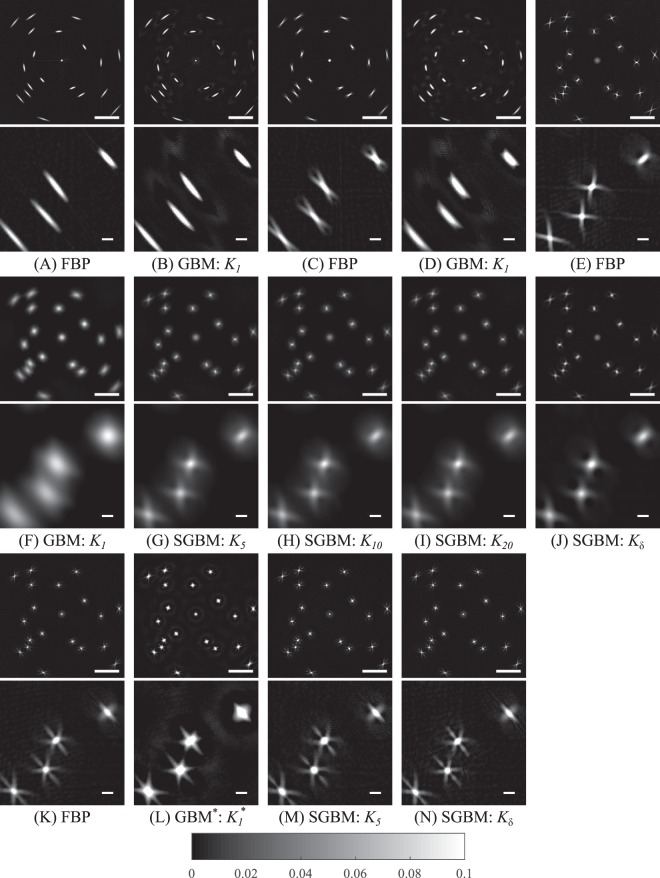
The reconstructions (**A**)–(**J**) obtained with the numerical phantom and simulated monofocal data. The region of interest shown below each reconstruction (rows 2 and 4) is from the bottom left part of the sample. Focal offset is 0 μm in (**A**) and (**B**), 75 μm in (**C**) and (**D**) and 225 μm in (**E**)–(**J**). The reconstructions (**K**)–(**N**) obtained with the numerical phantom and simulated multifocal data. The region of interest shown below each reconstruction is from the bottom left part of the sample. The scalebar length is 500 μm in rows 1, 3 and 5 and 50 μm in rows 2, 4 and 6.

The relative error measure (REM) and total variation error (TVE) values of (A)–(J) are presented in Table 2 and visualized in Fig. 2. The reconstructions (A)–(J) are shown in Fig. 3. With respect to REM, FBP was found to perform generally better than GBM. In both FBP and GBM, the reconstructed particles were blurred tangentially corresponding with the projection directions parallel to the light beam. The inversion errors increased as the offset was increased from 0 *μ*m to 75 *μ*m and further to 225 *μ*m. For these offsets, the REM of GBM was 1.6 and 2.6 times that of the no-offset case, and the REM of FBP was 1.3 and 4.9, respectively. TVE, on the contrary, did not provide a clear difference between the monofocal approaches. As the focal offset was increased, the overall contrast of the reconstruction and tangential blurring decreased, but FBP yielded more prominent streaking artifacts as seen in cases (A) to (C) to (E). Using the SGBM, a trade-off between roundness and smoothing was achieved. The REM decreased, and the contrast of the reconstructions increased along with the stretching, as seen in Table 2. However, the shape of the particles seems to have been slightly better maintained with moderate stretching.

**Table 2:** The numerical errors of the reconstructions (A)–(J) obtained with the numerical phantom and simulated Data.

ID	Focal offset	Inversion kernel	Dynamic range		REM (%)	TVE	Data acquisition mode
			min	max			
(A)	0 μm	FBP	−0.01	0.82	180	323.0	monofocal simulations
(B)	0 μm	*K*_1_	−0.07	1.13	440	778.1	
(C)	75 μm	FBP	−0.01	0.33	229	323.0	
(D)	75 μm	*K*_1_	−0.05	0.42	723	774.3	
(E)	225 μm	FBP	−0.04	0.25	888	323.0	
(F)	225 μm	*K*_1_	−0.00	0.04	1157	387.7	
(G)	225 μm	*K*_5_	0.00	0.07	721	403.2	
(H)	225 μm	*K*_10_	0.00	0.08	652	405.5	
(I)	225 μm	*K*_20_	0.00	0.08	622	406.6	
(J)	225 μm	*K*_*δ*_	0.01	0.13	463	432.0	
(K)		FBP	0.01	0.29	246	323.0	multifocal simulations
(L)		${K}_{1}^{\ast }$	−0.03	0.46	404	806.8	
(M)		*K*_5_	−0.02	0.38	428	754.4	
(N)		*K*_*δ*_	−0.02	0.37	350	862.8

In the multifocal simulations, the reconstructions (K)–(N) presented in Fig. 3 had particles with less elongation and with less prominent streaking. In terms of the REM values presented in Table 2, the reconstruction approaches GBM^*^ (L), SGBM (M) and (N) and FBP (K) were all roughly of the same order, although FBP provided the lowest REM and GBM^*^ the best contrast. Moreover, compared with the monofocal REM values, multifocal reconstructions were on the lower end of the error value scale. The TVE values, however, were relatively high for reconstructions from multifocal data.

### Results with experimental, monofocal data from the zebrafish

**Figure 4:**
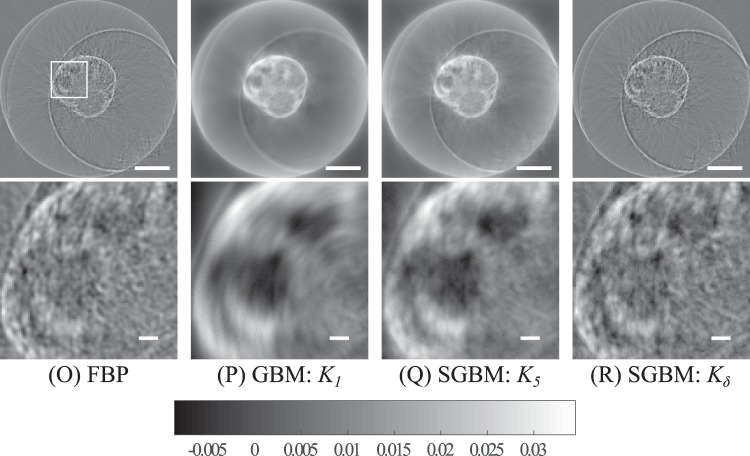
The reconstructions (O)–(R) obtained with the experimental zebrafish embryo sample and monofocal data and limited to the regions of interest. The location of the region of interest are marked in the top-left image. Scale bar in upper row is 250 μm and in lower row 25 μm. Region of interest has a width and height of 250 μm.

The reconstructions (O)–(R) of the experimental monofocal data from the zebrafish embryo are presented in Fig. 4. As shown by the visual analysis of region of interest, the GBM reconstruction (P) provided the best contrast, but was smoother than any of the other methods. In the case of SGMB using the kernel *K*_5_ (Q), a balance between tangential blurring was achieved, but with a subsequent decrease in contrast. FBP (O) and the Radon kernel *K*_*δ*_ SGBM (R) provided very similar reconstructions with the sharpest details of (O)–(R), but with also imminent streaking and halo artifacts.

### Results with experimental, multifocal data from the bead sample

**Figure 5:**
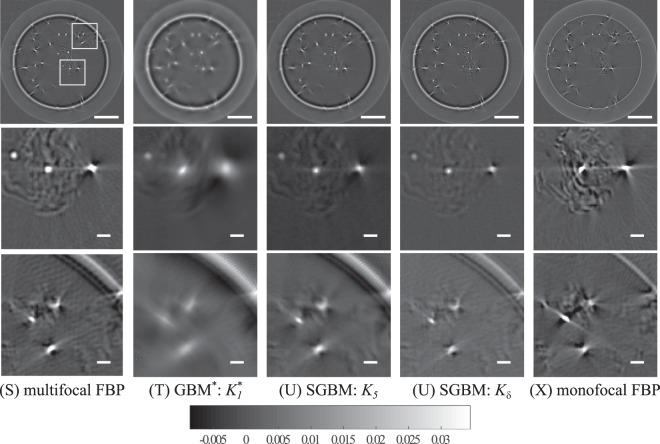
The reconstructions (S)–(V) obtained with the experimental bead phantom and multifocal data. (X) For comparison, the reconstruction obtained with the experimental bead phantom and monofocal data. The regions of interest are marked in image (S) and have a width and height of 500 μm. The scale bar length in row 1 is 500 μm and 50 μm in rows 2 and 3.

In Fig. 5, the multifocal (S)–(V) and monofocal (X) reconstructions are compared using the experimental data of the bead sample. Consistent with the above reported results of multifocal simulations, the multifocal data provided more details in the reconstructions in all cases. When compared with the monofocal FBP reconstruction (X), some particles were reconstructed only from the multifocal data. SGBM reconstruction (U) with the kernel *K*_5_ provided the same effect of trade-off as in monofocal case. Reconstruction (U) is sharper than blurry GBM* (T), and also shows less streaking and halo artifacts around the particles. Additionally, (U) has a smoother background compared with the Radon inversions FBP (S) and *K*_*δ*_ SGBM (V).

### Computational times and memory consumption

Computing one reconstruction of the TV-regularized iteration using a single processor thread took approximately 7 minutes CPU time and required 9.7 GB of RAM. With parallel computing using the Lenovo P910 workstation, computing times were in the order of 10 to 60 seconds, mainly depending on the used kernel. Using the present hard-thresholded SGBM approach, the memory consumption during the inversion stage is 5 to 15 times that of the plain inverse Radon transform. However, with GBM, the relative system size is 60 to 100 times that of the plain inverse Radon transform.

## Discussion

This study focused on the mathematical modeling of the brightfield version of optical projection tomography (OPT). We introduced the stretched Gaussian beam model (SGBM) and investigated its effect on imaging accuracy and artifacts. SGBM uses an elongated blurring kernel compared with the conventional Gaussian beam model (GBM) in order to provide more robustness with respect to forward errors and measurement noise. Additionally, similar to GBM, it also enables the exploitation of a larger projection aperture than the classical model of one-dimensional light beams. SGBM was evaluated with both numerical and experimental data as well as for both monofocal and multifocal measurements. As reconstruction techniques, we used total variation regularized (TV) iterative solutions for GBM and SGBM. We compared these reconstructions with filtered backprojection (FBP) computed reconstructions.

SGBM was found to be an efficient and robust surrogate for the GBM with significantly lower memory consumption, which enabled computations for larger image resolutions. The SGBM with the kernel *K*_5_ seemed to provide a sought-after trade-off between the image sharpness and the reconstructed object shape, when the forward modeling errors were large. This was observed to be the case for the large 225 μm focal offset in numerical experiments (E)–(J) and, especially, with the experimental data of the bead sample for which the reconstruction (T) based on GBM were overly smooth. Based on the region of interest comparisons, the streaking artifacts and halo effects were reduced in the SGBM reconstructions (Q) and (U) using *K*_5_ when compared with the respective FBP reconstructions (O), (S) and (X) or *K*_*δ*_ reconstructions (R) and (V). Preliminary tests with alternative regularization parameter values suggested that some artifacts could also be diminished by changing the regularization parameter values, but the major structural differences observed between the methods remained the same.

In addition to the gains in computational efficiency, a further motivation to use SGBM instead of GBM is the uncertainty related to the model parameters. In practice, the accurate positioning of the focal plane to the center of the sample is difficult if there is no reference point in the center of the sample. In this study, we tested several values of stretching factor *c*_*S*_ and of the focal offset. The results indicate, that the choice of *c*_*S*_ seems to be stable as the difference between *K*_5_, *K*_10_ and *K*_20_ was quite small in simulations in terms of relative error measure (REM) and total variation error (TVE).

The monochromatic light beam assumption used in this study included some errors because the Rayleigh range varies according to the wavelength of the light. We used only a single wavelength of 600 nm in our computations, but in reality, the white LED light has a spectrum in the order of 400 to 750 nm, which affects the Rayleigh length by up to ±25%. The GBM might benefit from having a spectral kernel, but in the case of SGBM, the elongation of the kernel already decreases the effect of spectral mismatch.

In multifocal imaging, a higher numerical aperture (NA) objective can be used to decrease the focal spot size, and thereby improve resolution close to the focal spot^30^. The cost of this is an increased overall level of blurring. However, to tackle the blurring, several methods can be employed to create a composite image with only focused details. In this study, we applied averaging which we consider a valid, although not necessarily the best, approach for multifocal data^32,46,47^. Alternatively, a so-called all-in-focus projection image can be formed by combining the focal parts of multiple projection images for a single angle and different focal plane distances. The final reconstruction can then be obtained via a standard inversion approach, such as FBP. Examples of all-in-focus fusion methods include^3,48,49^. In this study, we applied multifocal imaging without changing the objective, and hence the same NA throughout. The reconstructions computed from multifocal data provided finer details than reconstructions from monofocal data in all cases, even with the simplifying assumptions regarding creation of the composite image and the choice of objective.

A recent study compared several reconstruction techniques in fluorescence OPT^50^. They concluded that a reconstruction method based on point spread function analysis, which GBM and SGBM are, is good for nonsparse objects, such as the zebrafish embryo. On the other hand, for sparse objects, such as the bead sample, a different approach based on deconvolution produced better results. Our results seem to indicate that same might hold in brightfield OPT as well: visually more benefits are seen using GBM or SGBM with the zebrafish embryo when compared with the bead sample.

For practical applications, further quantitative verification is required and also motivated by the results of this study. The regularization weights in our reconstruction were minimal to make the effects of GBM and SGBM visible. In practice, moderately higher regularization should be applied, especially with the SGBM since visible streaking artifacts and halos were present in the TV reconstructions. The artifacts occurred because the optical and linear beam projections for a given image detail did not match in the directions where optical the beam was out of focus. Therefore, without an enhanced beam model, the OPT has a similarity to a limited-angle or sparse data imaging problem (see, e.g.^39^). Consequently, in addition to the regularization, the applied beam model was found to be essential to reduce the artifacts. The beam model might also turn out a valuable addition for upgrades of existing instrumentation, such as presented in^51^, where non-telecentric performance of low magnification lenses may turn out problematic. Furthermore, implementation of the blurring kernel in three dimensions would be a natural continuation of this work. This might, however, be difficult due to the already relatively large 2D system sizes obtained in this study. For example, inversion of the multifocal model with three different focusing kernels was not possible due to memory constraints. Therefore, it will be necessary to develop methods other than hard thresholding to compress the kernel.

Using different NA objectives, also the balance between actual lateral imaging accuracy and numerical compensation of the longitudinal resolution should be studied. To date, mostly low NA objectives with higher longitudinal resolution have been used. However, appropriate modeling, as shown here, will enable the use of higher NA objectives and take advantage of their higher lateral resolution.

## Conclusions

We have reported the use of Gaussian light beam model (GBM) in the reconstruction process of brightfield OPT. Using simulations, we studied the effect of focal offset, i.e., the difference of rotational center to the focal plane along the propagation axis. We found that the model was more robust to the focal offset via stretching the Rayleigh range artificially. This stretched Gaussian beam model (SGBM) was further tested with experimental data where, similarly to the simulations, the standard GBM produced overly smooth reconstructions while SGBM was able to balance between sharp details and a lower number of streaking artifacts.

## Materials and Methods

### Radon transform and blurring functional

**Figure 6:**
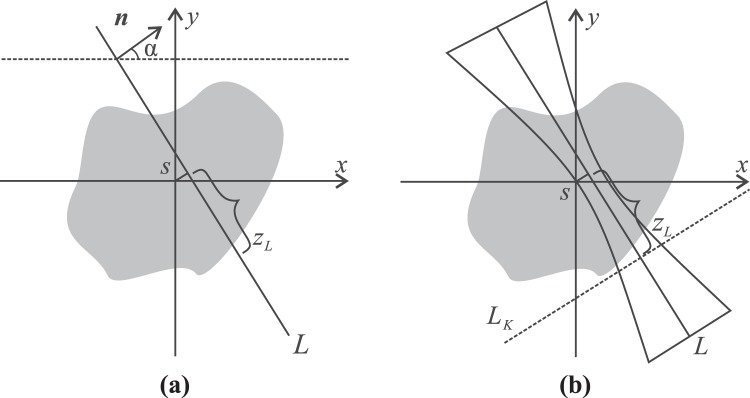
Schematic images for the Radon transform and the related Gaussian beam model (GBM). (**a**) The Radon transform integrates its target distribution along the line *L*. (**b**) The GBM describes a blurring process corresponding to an integral along the line *L*_*K*_ perpendicular to *L*. The hourglass-shaped contours illustrate the blurring kernel of the Gaussian beam.

The standard Radon transform^52^ ${\mathcal R} :{{\mathbb{R}}}^{2}\to {\mathbb{R}}$ is a line integral of a target function *f* along a given line *L*. Points (*x*, *y*) along *L* can be parametrized as

1 $$\begin{array}{ccc}x({z}_{L}) & = & {z}_{L}\,\sin \,\alpha +s\,\cos \,\alpha \\ y({z}_{L}) & = & -{z}_{L}\,\cos \,\alpha +s\,\sin \,\alpha \end{array}$$

where *s* is the distance of *L* from the origo, *α* is the angle between normal *n* of *L* and *x*-axis, and *z*_*L*_ is the arclength along *L*. The geometry of the Radon transform is presented in Fig. 6(a). With respect to the pair (*α*, *s*), the Radon transform of the function $g:{{\mathbb{R}}}^{2}\to {\mathbb{R}}$ is given by

2 $$ {\mathcal R} g(\alpha ,s)={\int }_{-\infty }^{\infty }f(x({z}_{L}),y({z}_{L}))d{z}_{L}.$$

The function *f* typically represents the image to be reconstructed. In this study, we consider *f* to be a blurred image of the form $f\mapsto { {\mathcal B} }_{K}f$, where ${\mathcal B} \,:\,{{\mathbb{R}}}^{2}\times {\mathbb{R}}\to {{\mathbb{R}}}^{2}$ is a linear blurring operator following from the GBM, similarly as already introduced to florescence OPT^38^. The source space of ${ {\mathcal B} }_{K}$ is composed by the Cartesian product ${{\mathbb{R}}}^{2}\times {\mathbb{R}}$ between the image space and the viewing angle. The operator ${ {\mathcal B} }_{K}$ is determined by a parameter we call blurring kernel $K:{{\mathbb{R}}}^{2}\to {\mathbb{R}}$, i.e., a function from the image space to a light intensity distribution. Illustrated in Fig. 6(b), the kernel is an axi-symmetric function in the coordinates (*x*_*L*_, *z*_*L*_) in which *x*_*L*_ is the distance to *L*. The kernel can also be interpreted as the shape of the individual light beams *L* propagating through sample. The focal spot of the beam, *z*_*L*_, is the position of the narrowest part of *L*. The blurring increases along with the value of |*z*_*L*_|.

In Fig. 6(b), the blurring kernel and the Radon transform are superimposed. The line *L*_*K*_ is orthogonal to *L*, and it intersects *L* at *z*_*L*_. In the coordinates (1), the blurred image $g={ {\mathcal B} }_{K}f$ is given by

3 $$({ {\mathcal B} }_{K}f)(\alpha ,s,{z}_{L})={\int }_{-\infty }^{\infty }\,K(\tau ,{z}_{L})f({x}_{K}(s-\tau ),{y}_{K}(s-\tau ))d\tau ,$$

where (*x*_*K*_(*z*_*K*_), *y*_*K*_(*z*_*K*_)) are the coordinates along line *L*_*K*_ and can be expressed similar to (1) as follows:

4 $$\begin{array}{ccc}{x}_{K}({z}_{K}) & = & {z}_{K}\,\cos \,\alpha +{z}_{K}\,\sin \,\alpha \\ {y}_{K}({z}_{K}) & = & -{z}_{K}\,\sin \,\alpha +{z}_{K}\,\cos \,\alpha .\end{array}$$

The blurring operator described in Eq. (3) is a convolution between the blurring kernel *K* and the image *f* computed at the point (*x*(*z*_*L*_), *y*(*z*_*L*_)). In other words, for each point in line *L*, the image function *f* is convoluted orthogonally along the line *L*_*K*_ with respect to the blurring kernel *K*.

To have a physical meaning, the blurring kernel $K(\tau ,z)\in {{\mathbb{R}}}^{2}$. corresponds to the normalized intensity profile of an axi-symmetric Gaussian beam^23,24^ and it is given by

5 $$K(\tau ,{z}_{L})=\sqrt{\frac{2}{\pi w({z}_{L})}}\exp (-\frac{2{\tau }^{2}}{w({z}_{L})})$$

with

6 $$w({z}_{L})={w}_{0}(1+{(\frac{{z}_{L}-{z}_{0}}{{z}_{{\rm{r}}}})}^{2}).$$

here, *z*_*L*_ is the axis of the propagation and *τ* is the radial distance from the *z*_*L*_-axis. The focal spot is placed at *z*_0_, and *w*_0_ is the width of the beam waist, i.e., the beam width at the focal spot. The Rayleigh range is *z*_r_ = *πw*_0_^2^/*λ*, where *λ* is the wavelength of the beam. The Rayleigh range and numerical aperture (NA) of the objective are related through NA = *w*_0_/*z*_r_. NA describes the focusing properties of the objective.

### Stretched gaussian beam model

To reduce the forward modeling errors related to practical detection of the focal plane, we introduce the stretched Gaussian beam model (SGBM) in which the Rayleigh range *z*_r_ is stretched, i.e., substituted with an expanded value *z*_r_ → *c*_*S*_*z*_r_ with *c*_*S*_ ≥ 1.

The SGBM can be interpreted as a surrogate forward approach in which the beam width is narrower when compared to the exact GBM. The motivation for using the SGBM is the ill-conditioned nature of the image deblurring task, because the SGBM allows the use of beam specific parameters in the forward model while ensuring that errors, such as the focal offset, will not be amplified in the inversion process.

The blurring kernel stretched by the factor *c*_*S*_ is denoted with ${K}_{{c}_{S}}$. For *c*_*S*_ = 1, the beam is non-stretched, i.e., *K*_1_ = *K*. Furthermore, for *c*_*S*_ → ∞ the beam approaches an intensity profile that is constant in the direction of propagation. If *w*_0_ → 0 simultaneously with *c*_*S*_ → ∞, then *K*_∞_ → *δ* with *δ* denoting the Dirac’s delta^53^. We denote the case *K* = *δ* with *K*_*δ*_ and it corresponds to no blurring, i.e., the standard Radon transform.

As previously mentioned and illustrated in Fig. 1, the choice of value for *c*_*S*_ depends on knowledge of the imaging system. In this paper, we assume that the shape of the detected beam is several orders more accurate than the focusing distance, i.e., the values of NA, *λ* and *w*_0_ depend on the components of the system, and uncertainty of their values is not discussed here. Instead, the placement of the sample, and thus the focusing distance *z*_0_ depends on the operator of the image acquisition, and its value is more prone to errors.

In the Gaussian model, the width of the beam is described by the Eq. 6 with respect to propagation coordinate *z*. Including the stretching of the Rayleigh range *z*_*r*_, we can use the Eq. 6 to formulate an expression to the stretching factor *c*_*S*_, which is

7 $${c}_{S}=\sqrt{\frac{{(\frac{z-{z}_{0}}{{z}_{r}})}^{2}}{\frac{w(z)}{{w}_{0}}-1}}.$$

In Eq. 7, the expression *z* − *z*_0_ can be used to estimate the discrepancy between of true, unknown focal distance and the range by which the focal distance is assumed to vary. For example, let us consider the case where the desired focal distance is in the center of the sample (*z*_0_ = 0), and we assume that the furthest possible true placement has a maximum 225 *μ*m offset, yielding *z* − *z*_0_ = 225 *μ*m. For NA = 0.14 and *λ* = 600 nm, the Rayleigh range has the value *z*_*r*_ = 9.7 *μ*m. To have the maximum weight on the reconstruction on the focal plane at the extreme offset, the beam width of the stretched beam at *z* = 225 *μ*m should match *w*_0_, in which case *c*_*S*_ → ∞ since *w*(*z*) = *w*_0_ (this is natural, since *w*(*z*) was supposed to be of Gaussian shape and stretching is trying to force a straight line, i.e., the Radon transform). Thus, the relation $\frac{w(z)}{{w}_{0}}$ describes the weighting of the uncertainty. A smaller fraction means more stretching, and that the focal offset is assumed to be in the extreme of its supposed range. A higher fraction, i.e., less stretching supposes that the real focal offset is likely to be close to *z*_0_ = 0. Further, assuming that the focal offset is likely closer to the extreme offset; and weighting the extreme offset to be quite likely and thus allowing the stretched offset to only double the initial beam width at *z*_0_, that is, *w*(225 *μ*m) = 2*w*_0_; stretching factor should have the value of *c*_*S*_ ≈ 23.2 Similarly, the choice of *w*(225 *μ*m) = 7*w*_0_ yields *c*_*S*_ ≈ 9.5, or the choice of *w*(75 *μ*m) = 2*w*_0_ gives *c*_*S*_ ≈ 7.7.

### Forward operator and inversion algorithm

Our forward operator describes the image information collected by the lens from the light propagating through the sample. The forward operator combines the Radon transform ${\mathcal R}$ with the GBM and SGBM via the blurring functional ${ {\mathcal B} }_{K}$. In the following analysis, we additionally assume that the light beams are parallel. The measurement (projection) data *y* for a given image *I*, is assumed to obey the following linear model with an additive noise term *n*:

8 $$y= {\mathcal R} \circ { {\mathcal B} }_{K}I+n={\int }_{-\infty }^{\infty }\,{\int }_{-\infty }^{\infty }\,Kf\,d\tau \,d{z}_{L}+n.$$

We emphasize that the operators ${\mathcal R}$ and ${\mathcal B}$ cannot be commuted — blurring depends on the depth, but the Radon transform suppresses information in the direction of the light propagation. Hence, physically interpreted, it is a single operator that describes the portion of light propagation collected by the lens. If *K*(*τ*, *z*) = *δ*(*τ*) with *δ* denoting the Dirac’s delta, then the operator of (8) corresponds to the Radon transform, i.e., ${\mathcal R} f= {\mathcal R} \circ { {\mathcal B} }_{K}f$.

For reasons of practical memory consumption, the kernel entries below a given (hard) threshold value were set to zero in all computations. We used a threshold value of *e*^−2^${K}_{{c}_{S}}$ (0, *z*_*L*_), i.e., *e*^−2^ times the on-axis value along propagation.

To compute a reconstruction, we use the following iteration:

9 $${f}_{\ell +1}={({{\rm{L}}}^{T}{\rm{L}}+{{\rm{D}}\Gamma }_{\ell }{\rm{D}})}^{-1}{{\rm{L}}}^{T}y$$

in which $y\in {{\mathbb{R}}}^{m}$, $f\in {{\mathbb{R}}}^{n}$, ${\Gamma }_{\ell }={\rm{diag}}\,({\gamma }_{1},{\gamma }_{2},\,\ldots ,\,{\gamma }_{n})$ with ${\gamma }_{i}={|{\rm{D}}f|}_{i}^{-1}$, and ${\rm{D}}$ is a regularization matrix given by

10 $${{\rm{D}}}_{i,j}=\frac{{\beta }_{1}(2{\delta }_{i,j}-1)\,{\int }_{{{\rm{P}}}_{i}\cap {{\rm{P}}}_{j}}\,{\rm{d}}s}{\mathop{{\rm{\max }}}\limits_{i,j}\,{\int }_{{{\rm{P}}}_{i}\cap {{\rm{P}}}_{j}}\,{\rm{d}}s}+{\beta }_{2}{\delta }_{i,j},\,{\rm{and}}$$

11 $${\delta }_{i,j}=\{\begin{array}{ll}1, & {\rm{if}}\,j=i\\ 0, & \mathrm{otherwise}\,{\rm{.}}\end{array}$$

Here, P_*i*_ denotes the *i*-th image pixel. The first term penalizes the jumps over the edges and the second one the norm of *f* and *β*_1_ and *β*_2_ are the regularization parameters, i.e., weights given for the respective terms. If this iteration converges, it minimizes the regularized objective function

12 $$F(x)={\Vert {\rm{L}}x-y\Vert }_{2}^{2}+2{\Vert {\rm{D}}x\Vert }_{1}$$

in which the latter norm is the total variation of *x*, if *β*_2_ = 0^39,40^. We used a low value for *β*_2_ to ensure the invertibility of ${\rm{D}}$ and thereby the converge of the algorithm. ${\rm{L}}$ is the matrix formulation of the combined transform ${\mathcal R} \circ  {\mathcal B}$, formed by inserting the Gaussian shape along each detection line. A detailed analysis of the algorithm is presented in^39^. The MATLAB code used in this article is provided at^54^ with example data. Another, complete data set imaged by us is available at^55^.

### Numerical experiments

**Figure 7:**
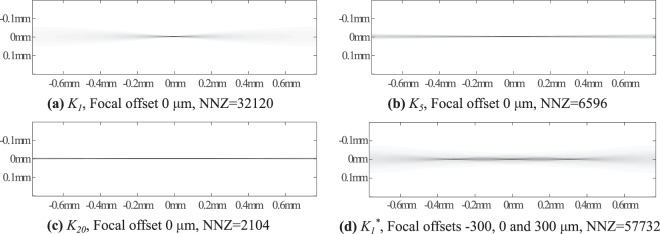
(**a**) The Gaussian beam kernel *K* corresponding to NA = 0.14 and *λ* = 600 nm. (**b**) Extended kernels *K*_5_ and (**c**) *K*_20_. (**d**) A multifocal kernel K^*^ = (*K*^(1)^ + *K*^(2)^ + *K*^(3)^)/3 in which NA = 0.14 and *λ* = 600 nm and the focal offsets of *K*^(1)^, *K*^(2)^ and *K*^(3)^ are −300, 0 and 300 *μ*m, respectively. NNZ is the number of non-zero elements in the kernel. Horizontal axis is the propagation axis *z*_*L*_ and vertical axis is the radial distance *τ*.

In estimating the blurring kernels, we approximated the brightfield with a single wavelength *λ* = 600 nm in air, further adjusted for sample immersion liquid, in this case water with a refractive index of 1.33. The Rayleigh range had the value *z*_*r*_ = 9.7 *μ*m following from the experimental setup objective of NA = 0.14. For forward solution, we used standard Gaussian kernel *K*_1_ with the focal offsets 0, 75, and 225 *μ*m. Zero-offset *K*_1_ is shown in Fig. 7(a). In order not to commit an inverse crime, the forward solutions in the simulations were computed with non-thresholded *K*_1_. All of the inverse solutions were computed using thresholded kernels: *K*_1_ without focal offset for GBM, and *K*_5_, *K*_10_, *K*_20_ and *K*_*δ*_ for SGBM. *K*_5_ and *K*_20_ are shown in Fig. 7(b,c), respectively. For comparison, FBP^52^ reconstructions were computed using MATLAB’s built-in function iradon with Hamming filter. Figure 7 also includes the number of non-zero elements of the kernels as they relate directly to the system inverted system sizes.

In multifocal experiments, the projection data of each angle was averaged into a single projection for the inversion. The multifocal kernel is referred to as GBM^*^, and it consists of averaging three *K*_1_ kernels with different offsets, i.e., ${K}_{1}^{\ast }=(1/N)\mathop{\sum }\limits_{i=1}^{N}\,{K}^{(i)}$ where *K*^(1)^, *K*^(2)^, *K*^(3)^ of the individual focal offsets *i* = 1, 2, 3. The multifocal kernel K^*^ is shown in Fig. 7(d). For reconstructions in simulated cases, we used the GBM with kernel *K*_1_ without focal offset, and the SGBM with kernels *K*_5_, *K*_10_, *K*_20_ and *K*_*δ*_; and FBP. The kernels *K*_1_, *K*_5_ and *K*_20_ are shown in Fig. 7.

**Figure 8:**
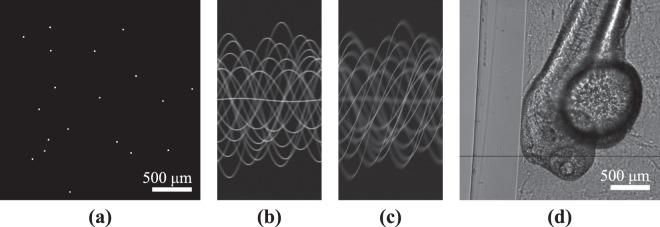
(**a**) The numerical 512 × 512 phantom with the side length of 1 mm and particle diameter of 10 μm. (**b**) The numerical sinogram without focal plane offset and (**c**) with 225 μm offset in kernel *K*_1_. (**d**) A projection image from the zebrafish embryo data set. Reconstruction shown in this article are from a height of 1600 (pixel row illustrated with a black line). Image contrast has been adjusted for printability.

Figure 8(a) shows our numerical phantom that was a square of 512 × 512 pixels with a side length corresponding to 1 mm and particle diameter to 10 μm (approximately 19 pixels). The background had the attenuation coefficient of zero (i.e., that of the void) and the particles had the attenuation coefficient of one. The numerical projection data comprised 400 projections in the range [0.9°, 360°] with 0.9° angular increment. Projection data were simulated using kernels *K*_1_ with no focal offset (shown in Fig. 8(b) and with 75 and 225 μm focal offsets (the 225 μm offset data are shown in Fig. 8(c)). In total, we present 15 simulated studies labeled with the letters (A)–(N) and combinations of these are presented in Table 1 showing the forward and inverse parameters used.

As the measurement noise, we used additive normal distributed noise with standard deviation 0.01 relative to the maximal noiseless data entry. For the GBM and SGBM reconstructions, we used three iteration steps. After trying different iteration lengths up to ten steps, three steps were found to be sufficient.

The numerical accuracy was analyzed via the relative error measure (REM) which we defined as the relative 1-norm ${\Vert f\Vert }_{1}=\int |f|\,\,dxdy$ between the phantom and the reconstruction *f*_rec_ with its minimum shifted to zero and maximum scaled to one. We expressed REM in percentages as ${\rm{REM}}=100\frac{{\Vert {f}_{{\rm{relative}}}-{f}_{{\rm{phantom}}}\Vert }_{1}}{{\Vert {f}_{{\rm{phantom}}}\Vert }_{1}},$ where ${f}_{{\rm{relative}}}=\frac{{f}_{{\rm{rec}}}}{{f}_{{\rm{\max }}}-{f}_{{\rm{\min }}}}-{f}_{{\rm{\min }}}$. The 1-norm was chosen because it is known to be small for sparse distributions with only a few non-zero values^56,57^, such as the cell phantom distributions used here. REM value measures the general quality of the image without penalizing the change in dynamic range. To analyze the dynamic accuracy, we evaluated the following relative total variation error (TVE):

$${\rm{TVE}}=100\,\frac{{\Vert {f}_{{\rm{rec}}}-{f}_{{\rm{phantom}}}\Vert }_{{\rm{TV}}}}{{\Vert {f}_{{\rm{rec}}}\Vert }_{{\rm{TV}}}}\,{\rm{with}}\,{\Vert f\Vert }_{{\rm{TV}}}=\int |\nabla f|\,{\rm{dxdy}}{\rm{.}}$$

### Experimental data

The experimental data were obtained from two experimental samples, one being a zebrafish embryo and the other composed of hydrogel with small polymer beads as cell phantoms. We used the in-house OPT^20,21^ where the projection images were captured using an sCMOS camera (ORCA-Flash 4.0, Hamamatsu, Japan) with an infinity-corrected long working distance objective lens of either 5X for bead sample (Edmund, USA, NA = 0.14) or 10X for zebrafish embryo (Edmund, USA, NA = 0.28). The instrumentation included an iris diaphragm (Thorlabs, USA) and a filter wheel (Thorlabs, USA) between the objective and the camera, but these were not in use for the data used in this article, i.e., the iris was fully open, and there was no filter in the filter wheel. The projection data of both samples were acquired with a 0.9° interval from 0° to 359.1°, 400 images in total. Before imaging, both samples were manually centered in the *x*-coordinate using horizontally symmetric rotation and along the *y*-coordinate using the tube walls as a visual aid.

The zebrafish embryo was a two days post fertilization Tg(fli:1a:eGFP) prepared in 1.5% agarose (Sigma Aldrichm, Finland) hydrogel with added Tricaine (Sigma Aldrichm, Finland) for anesthetizing the fish. From the hydrogel, the fish was then sucked into a fluorinated propylene ethylene (FEP) tube with a syringe. The FEP tube had an inside diameter of 2 mm. The sample was imaged after mounting using a 10X objective with an exposure time of 8 ms. Only one focal plane was used for this image set. A single projection from the zebrafish embryo data is shown in Fig. 8(d). The reconstructions are computed from a height (pixel row) of 1600.

The keeping and raising of zebrafish stocks was performed with permission of the State Provincial Office of Western Finland (permission ESAVI/10079/04.10.06/2015). Care and experimental use of zebrafish embryo was carried out in accordance with EU Directive 2010/63/EU on the protection of animals used for scientific purposes; the Finnish Act on the Protection of Animals Used for Scientific or Educational Purposes (497/2013); and the Government Decree on the Protection of Animals Used for Scientific or Educational Purposes (564/2013).

The bead sample was prepared from gellan gum (GG) with physical crosslinking via 0.6% (w/w) spermine crosslinker, both dissolved in a 10% (w/v) sucrose solution (Sigma Aldrichm, Finland)^58^. Spherical, black polystyrene beads (0.5% v/v) with a 10 *μ*m diameter (Polysciences, USA) were mixed in the hydrogel during gelation. For the image acquisition, the sample was placed inside a FEP tube, which had an inside diameter of 2 mm. Two additional acquisitions were performed after translating the sample to 600 nm either closer or further to the objective lens than the original placement. The multifocal data were then processed by averaging through all three projections in each angle. An exposure time of 6.50 ms and 5X objective were used in imaging.

For computations, the data were linearized by scaling the values to interval [10^−10^, 1] and taking the 10-based logarithm (−log_10_(·)). The center of rotation was corrected manually by shifting the sinogram with different offsets and choosing the offset with visually the least amount of circular effects caused by center of rotation offset^42,44^. For memory reasons, the reconstructions were computed on a 512 × 512 pixel grid, and hence the width of the sinogram was average downscaled to 512 pixels from the original 2048.

From the experimental data, we present nine reconstructions labeled with the letters (O)–(X) the details of which are presented in Table 1. The reconstructions (O)–(R) from the monofocal data of the zebrafish embryo were computed using FBP, GBM with kernel *K*_1_ and SGBM with kernels *K*_5_ and *K*_*δ*_. Reconstructions (S)–(V) were computed from the multifocal data of the bead sample with FBP, GBM with kernel K^*^ and SGBM with kernels *K*_5_ and *K*_*δ*_. For comparison, one reconstruction (X) was computed from the bead sample with monofocal data using FBP. In GBM and SGBM reconstructions, we used three iteration steps. The values of *β*_1_ and *β*_2_ are shown in Table 1. In the SGBM cases, we report only *c*_*S*_ = 5 cases.

### Hardware and software used

The computations were performed using a high-end Lenovo P910 Workstation equipped with two Intel Xeon E5-2697 processors and 256 GB RAM. As the computation platform we employed MATLAB version R2016b 64-bit (The MathWorks, Inc.). The MATLAB scripts written for the blurring kernels, Radon transform and the inversion routine (9) have been included in^54^.

## Data Availability

MATLAB codes and data related to this article are shared through Zenodo at 10.5281/zenodo.1469361.
